# Identification and functional characterization of prognosis-related ferroptosis-associated lncRNAs in colorectal cancer

**DOI:** 10.3389/fimmu.2025.1561210

**Published:** 2025-04-29

**Authors:** Xiaoxu Ge, Jiasheng Xu, Jinjie He, Jian Wang, Yucheng Qian

**Affiliations:** ^1^ Department of Colorectal Surgery and Oncology, Key Laboratory of Cancer Prevention and Intervention, China National Ministry of Education, The Second Affiliated Hospital, Zhejiang University School of Medicine, Hangzhou, Zhejiang, China; ^2^ Department of Colorectal Surgery and Oncology, Key Laboratory of Molecular Biology in Medical Sciences, The Second Affiliated Hospital, Zhejiang University School of Medicine, Hangzhou, Zhejiang, China; ^3^ Center for Medical Research and Innovation in Digestive System Tumors, Ministry of Education, The Second Affiliated Hospital, Zhejiang University School of Medicine, Hangzhou, Zhejiang, China; ^4^ Zhejiang Provincial Clinical Research Center for Cancer, The Second Affiliated Hospital, Zhejiang University School of Medicine, Hangzhou, Zhejiang, China; ^5^ Cancer Center of Zhejiang University, The Second Affiliated Hospital, Zhejiang University School of Medicine, Hangzhou, Zhejiang, China; ^6^ Department of Gastroenterology, the Second Affiliated Hospital, School of Medicine and Institute of Gastroenterology, Zhejiang University, Hangzhou, Zhejiang, China; ^7^ Department of Thyroid Surgery, The Second Affiliated Hospital, Zhejiang University School of Medicine, Hangzhou, Zhejiang, China

**Keywords:** colorectal cancer, ferroptosis, lncRNAs, prognostic model, therapeutic targets

## Abstract

**Background:**

Colorectal cancer (CRC) is a significant global health burden, with current treatment strategies often limited by the TNM classification system’s inability to fully capture tumor heterogeneity. This study aims to explore the biological functions and prognostic value of differentially expressed ferroptosis-related long non-coding RNAs (DEFRlncRNAs) in CRC.

**Methods:**

We utilized the TCGA database to identify DEFRlncRNAs associated with CRC prognosis. Through multivariate Cox regression analysis, we constructed a prognostic model and selected three key lncRNAs: Lnc-SH2D3A-2, Lnc-LSS-1, and Lnc-PEX11G-4. We assessed their expression in CRC and normal colonic epithelial cell lines using qPCR. Further functional assays included ferroptosis induction with RSL3 and Erastin, cell viability assessments, immunofluorescence staining for lipid peroxidation, and Western blot analysis of ferroptosis-related proteins.

**Results:**

Our analysis identified 15 DEFRlncRNAs significantly associated with CRC prognosis, with Lnc-SH2D3A-2, Lnc-LSS-1, and Lnc-PEX11G-4 showing high basal expression in CRC cell lines. Knockdown of Lnc-LSS-1 and Lnc-PEX11G-4 in HT29 and DLD1 cells resulted in significant inhibition of ferroptosis induced by RSL3 and Erastin. The mechanism behind the suppression of ferroptosis by knockdown of Lnc-LSS-1 and Lnc-PEX11G-4 may involve the inhibition of lipid peroxidation and the upregulation of GPX4 expression.

**Conclusion:**

DEFRlncRNAs, particularly Lnc-LSS-1 and Lnc-PEX11G-4, play crucial roles in regulating ferroptosis in CRC. These lncRNAs have potential as novel prognostic markers and therapeutic targets, providing valuable insights for personalized CRC treatment strategies.

## Introduction

Colorectal cancer (CRC) is among the most prevalent malignancies of the digestive system. According to the latest global cancer burden statistics released in 2023, CRC continues to rank high in both incidence and mortality worldwide (1). The primary therapeutic strategies for CRC include surgery, chemotherapy, radiotherapy, targeted therapy, and immunotherapy. However, the conventional TNM (Tumor, Node, Metastasis) classification, which forms the basis of staging and treatment planning, has notable limitations. This system may not fully capture the biological diversity and complexity of colorectal cancer, and as a result, not all patients benefit from treatment plans strictly designed according to TNM staging. To address these limitations, molecular profiling of tumors has gained increasing importance in guiding therapeutic decisions. The Consensus Molecular Subtypes (CMS) classification, for instance, stratifies CRC into distinct molecular subtypes, providing critical insights into tumor biology and patient prognosis (2). This approach enhances the prediction of treatment responses and clinical outcomes, allowing for therapies to be more precisely tailored to individual patient profiles. Given these advancements, there is a pressing need to develop predictive models that integrate a broader range of clinical, pathological, and molecular data to improve diagnostic accuracy and optimize treatment strategies for CRC patients.

Ferroptosis is a distinct form of regulated cell death characterized by iron-dependent lipid peroxidation and subsequent cell membrane damage (3). Unlike apoptosis, necrosis, or autophagy, ferroptosis is driven by the accumulation of iron and reactive oxygen species (ROS), leading to oxidative stress and cell death. This process is predominantly regulated by the metabolism of glutathione and the enzyme glutathione peroxidase 4 (GPX4), which converts lipid hydroperoxides into non-toxic lipid alcohols. Inhibition of GPX4 or depletion of glutathione results in the accumulation of lipid peroxides, thereby triggering ferroptosis. Additionally, SLC7A11 plays a critical role in maintaining intracellular glutathione levels, and its inhibition lowers glutathione, promoting ferroptosis. Research has demonstrated that ferroptosis inducers like RSL3 (4) and Erastin (5) can effectively reduce the growth of human CRC cell lines, highlighting the potential of targeting ferroptosis as a therapeutic strategy for CRC treatment.

Long non-coding RNAs (lncRNAs), which are non-protein-coding transcripts exceeding 200 nucleotides in length, play various roles in chromatin remodeling, transcriptional regulation, and post-transcriptional processing (6). Recent studies have revealed the involvement of lncRNAs in multiple aspects of cancer biology, including tumor growth, metastasis, and resistance to therapy. In CRC, lncRNAs have been shown to regulate key signaling pathways that drive tumor progression and influence patient prognosis (7–10). Moreover, emerging evidence suggests that lncRNAs modulate ferroptosis, acting as either promoters or inhibitors of this form of cell death (11). By regulating the expression of genes associated with iron metabolism and oxidative stress, lncRNAs can influence ferroptosis and, consequently, impact tumor behavior. Understanding the roles of ferroptosis-related lncRNAs in CRC could lead to the identification of novel prognostic markers and therapeutic targets, offering valuable insights for developing personalized cancer treatment models.

Given the growing interest in the relationship between ferroptosis-related lncRNAs and CRC, this study aims to explore their potential prognostic value and shed light on their role in the disease.

## Methods

### Public databases

We obtained raw RNA-seq expression profiles and corresponding clinical information for colon adenocarcinoma (COAD) and rectum adenocarcinoma (READ) from The Cancer Genome Atlas (TCGA) database (https://portal.gdc.cancer.gov/). To identify gene sets associated with ferroptosis, we employed the Gene Set Enrichment Analysis (GSEA) platform. This involved selecting ferroptosis-related genes from the RNA-seq data using predefined gene sets available on GSEA. Subsequently, we used the STRING database (https://www.string-db.org/) to predict lncRNAs that interact with the ferroptosis-related genes identified in the previous step. STRING provides an extensive network of known and predicted protein-protein interactions, which we leveraged to infer potential lncRNA interactions with ferroptosis-related genes.

### Functional enrichment analysis

We utilized the Gene Ontology (GO) and Kyoto Encyclopedia of Genes and Genomes (KEGG) databases. GO analysis was conducted to identify enriched biological processes, cellular components, and molecular functions associated with the differentially expressed ferroptosis-related lncRNAs (DEFRlncRNAs). KEGG pathway analysis was used to explore the involvement of these lncRNAs in specific signaling pathways. These analyses were carried out using the “clusterProfiler” R package, which provided an overview of the potential roles of DEFRlncRNAs in ferroptosis-related cellular processes. Results were visualized using bar plots to highlight the most significantly enriched terms and pathways.

### Establishment of the risk signature

To identify DEFRlncRNAs associated with the prognosis of CRC patients, we first performed a univariate Cox regression analysis. Subsequently, a LASSO Cox regression analysis was employed to construct the prognostic model (12). This model was developed using a penalized maximum likelihood estimator, validated through 1000-fold cross-validation. The risk score for each patient was calculated using the following formula (13): $\text{Risk Score}=\;\sum_{i=1}^{n}\left(\beta_{i}\times Expression\;Level\;of\;Gene_{i}\right)$ . Based on the median risk score derived from TCGA data, patients were classified into low-risk and high-risk groups.

### Survival and ROC analysis

To evaluate the prognostic value of the risk score model, we conducted Kaplan-Meier (KM) survival and receiver operating characteristic (ROC) curve analyses. KM survival curves were generated to illustrate differences in survival between low-risk and high-risk groups, utilizing the “survival” and “survminer” packages in R. Statistical significance between these groups was determined using log-rank tests. The predictive accuracy of the risk score model over time was assessed with ROC analysis via the “timeROC” package (14) in R. We plotted ROC curves to evaluate the model’s ability to differentiate between risk groups at 1-year, 2-year, and 3-year survival intervals by comparing sensitivity (true positive rate) to 1-specificity (false positive rate) across various thresholds. The area under the ROC curve (AUC) was computed for each time point to measure the model’s discriminatory power, with an AUC closer to 1.0 indicating stronger predictive performance.

### Nomogram and decision curve analysis

To evaluate the prognostic value of our model, we constructed a nomogram that integrates significant clinical variables with the risk score. The nomogram was created using the “rms” package in R, providing a visual representation of the model’s predictive capability. Calibration curves were used to compare predicted outcomes with actual results. Decision curve analysis (DCA) was conducted using the “rmda” package to assess the model’s clinical utility by calculating the net benefit across a range of threshold probabilities, demonstrating its potential clinical impact compared to standard strategies.

### MCPcounter analysis

We applied the MCPcounter method (15) to estimate the abundance of various immune and stromal cell populations in CRC tissue. MCPcounter is a computational algorithm that uses transcriptomic data to quantify specific cell types. The analysis was performed using the “MCPcounter” package in R, which calculates cell-type-specific scores from gene expression data. These scores enabled us to evaluate the infiltration levels of different immune and stromal cells, offering insights into the tumor microenvironment.

### lncRNA ID conversion

We utilized the LNCipedia database (https://lncipedia.org/) to convert lncRNA IDs. The process involved accessing the LNCipedia website, entering the target lncRNA name in the search bar, and retrieving the corresponding lncRNA ID along with related annotation information.

### Quantitative polymerase chain reaction

Total RNA was extracted from cell lines using Trizol reagent (TAKARA) and reverse transcribed into cDNA (YEASEN) according to the manufacturer’s instructions. qPCR was performed using SYBR Green Master Mix (YEASEN). Specific primers for target genes were used. The relative gene expression was calculated using the 2^(-ΔΔCt) method, with GAPDH as the internal control. All reactions were run in triplicate and analyzed for statistical significance.

### Western blotting

Cells were treated with various concentrations and types of ferroptosis agonists for 48 hours, then harvested and lysed in NP40 buffer (Beyotime) with Protease Inhibitor Cocktail (Selleck). Protein concentrations were determined using the BCA assay (Pierce). Equal amounts of protein were separated by SDS-PAGE and transferred to PVDF membranes. Membranes were incubated with primary antibodies (all from CST, 1:1000) followed by goat anti-mouse/rabbit secondary antibodies (1:5000).

### siRNA-mediated gene knockdown

The siRNA and si-Ctrl sequences were purchased from RiboBio (Guangzhou, China), and the specific sequences are listed in **Supplementary Table S3**. Cells were transfected with siRNA targeting gene of interest using Lipofectamine 3000 (Invitrogen) according to the manufacturer’s protocol. Briefly, cells were seeded in 6-well plates and allowed to reach 30-50% confluence. siRNA and Lipofectamine 3000 were diluted in Opti-MEM (Gibco) and incubated for 15 minutes at room temperature. The siRNA-Lipofectamine complexes were then added to the cells and incubated for 72 hours. Knockdown efficiency was assessed by qPCR.

### Cell viability assay

Cell viability was assessed using the Cell Counting Kit-8 (CCK-8, YEASEN) according to the manufacturer’s instructions. Cells were seeded in 96-well plates at a density of 10,000 cells per well in 100 µL and cultured. After treatment with ferroptosis agonists for 48 hours, 10 µL of CCK-8 solution was added to each well and incubated for 1 hour at 37°C. Absorbance at 450 nm was measured using a microplate reader to calculate the inhibition rate of cell viability.

### Immunofluorescence staining

DLD1 cells were seeded onto coverslips in 24-well plates and allowed to adhere overnight, followed by stimulation with the ferroptosis agonist RSL (2.5 μM) for 48 hours. They were then fixed with 4% paraformaldehyde for 30 minutes at room temperature and permeabilized with 0.1% Triton X-100 for 10 minutes. After blocking with 5% BSA in PBS for 1 hour, cells were incubated with a lipid peroxidation-specific fluorescent probe (L267, DOJINDO) as per the manufacturer’s instructions. Nuclei were stained with DAPI for 5 minutes. Coverslips were mounted onto slides using anti-fade mounting medium. Fluorescent images were captured using a fluorescence microscope, with ROS levels quantified by measuring the fluorescence intensity of the ROS probe, and nuclear morphology assessed by DAPI staining.

### Statistical analysis

All statistical analyses were performed using R software (version 3.6.1, https://www.r-project.org/). Gene expression levels between tumor and normal tissue samples were compared using the Student’s t-test. Comparisons between multiple groups were performed using a one-way analysis of variance. For categorical variables, differences in proportions across groups were assessed with the chi-square test. Survival analysis was conducted through the Kaplan-Meier method, and statistical differences in overall survival (OS) between high-risk and low-risk groups were evaluated using the log-rank test. To identify factors independently associated with OS, univariate and multivariate Cox proportional hazards regression models were applied. A P value of less than 0.05 was considered statistically significant, P values are provided in the source data that accompanies this manuscript. Data are presented as mean ± s.e.m. and all tests were two-tailed unless otherwise specified.

## Results

### Identification of differentially expressed ferroptosis-related lncRNAs in CRC patients

**Figure 1:**
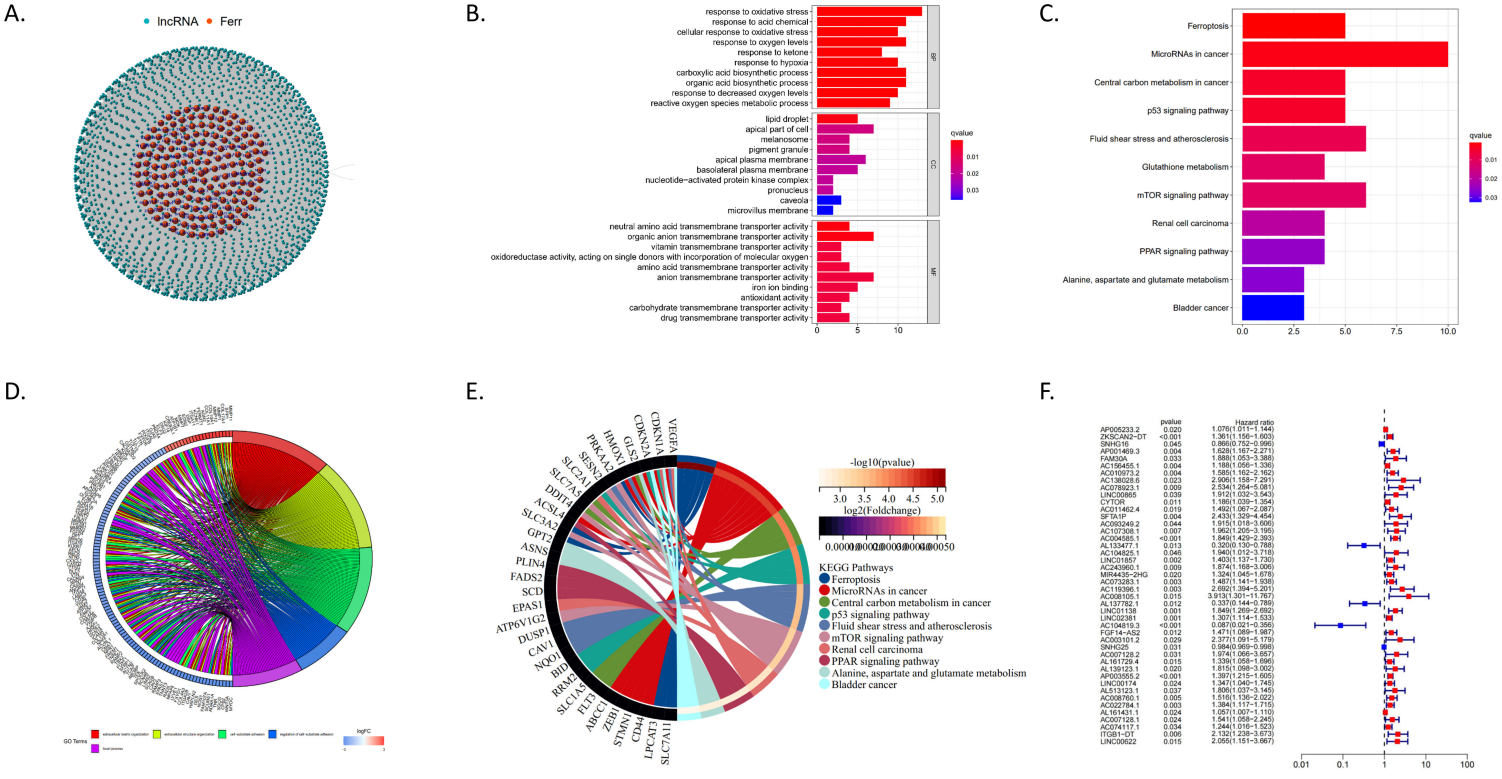
**(A)** Red dots represent 68 ferroptosis-related genes that are differentially expressed between CRC and normal tissues, while blue dots indicate 736 DEFRlncRNAs. **(B)** GO enrichment analysis results for DEFRlncRNAs. **(C)**. KEGG pathway analysis showing significant enrichment of DEFRlncRNAs. **(D)** The top 5 enriched GO pathways for differentially expressed genes in the high expression group. **(E)** The top 10 enriched KEGG pathways for differentially expressed genes in the high expression group. **(F)** Forest plot from univariate Cox regression analysis showing 44 DEFRlncRNAs correlated with CRC patient prognosis.

We identified 68 ferroptosis-related genes that were differentially expressed between CRC tissues and normal tissues using data from the TCGA database. Through the STRING database, we predicted interactions with these genes and ultimately identified 736 differentially expressed ferroptosis-related lncRNAs (DEFRlncRNAs, **Figure 1A**). Functional enrichment analysis of these DEFRlncRNAs revealed significant findings. Gene Ontology (GO) enrichment analysis showed that DEFRlncRNAs were mainly associated with the biological process “response to oxidative stress,” the cellular component “lipid droplet,” and the molecular functions “antioxidant activity” and “iron ion binding” (**Figure 1B**). Additionally, the Kyoto Encyclopedia of Genes and Genomes (KEGG) pathway analysis indicated significant enrichment in the ferroptosis process, p53 signaling pathway, glutathione metabolism, and mTOR signaling pathway, supporting the validity of our DEFRlncRNA selection (**Figure 1C**). We also observed enrichment in alanine, aspartate, and glutamate metabolism, as well as the PPAR signaling pathway (**Figures 1D, E**). Using univariate Cox regression analysis, we identified 44 lncRNAs from the DEFRlncRNAs that were correlated with CRC patient prognosis. The analysis revealed that the high expression of 39 lncRNAs, with the exceptions of SNHG16, AL133477.1, AL137782.1, AC104819.3, and SNHG25, was associated with poor prognosis in CRC patients (**Figure 1F**).

### Construction of a prognostic prediction model based on DEFRlncRNAs expression in CRC patients

**Figure 2:**
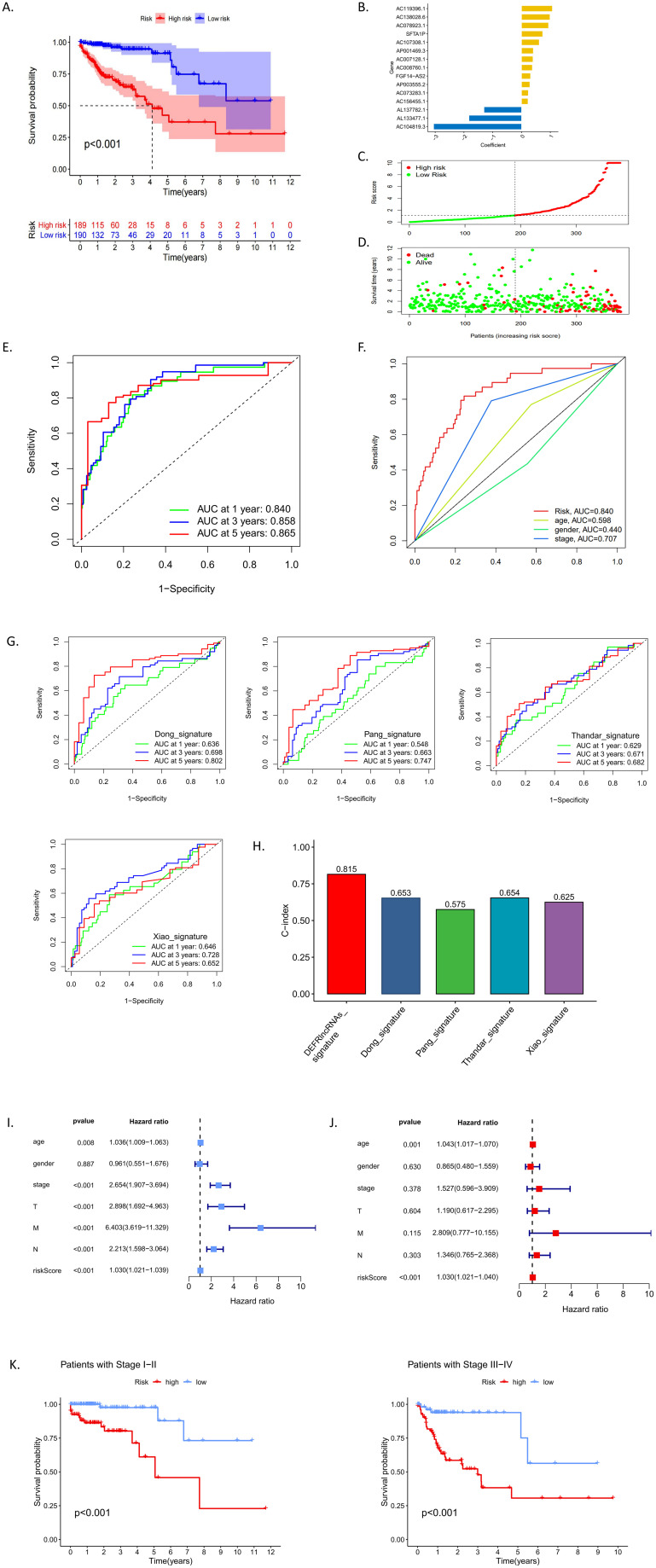
**(A)** Kaplan-Meier survival analysis comparing high-risk and low-risk groups classified by median risk scores. **(B)** A bar chart displaying the correlation coefficients of each DEFRlncRNAs that constituted the prognostic prediction signature. **(C, D)** Distribution of risk scores and corresponding survival status according to the prediction model. **(E)** ROC curves indicating the prognostic accuracy of the signature at 1, 3, and 5 years. **(F)** Comparison of ROC curves for clinical factors versus the risk model. **(G)** ROC curves illustrating the predictive accuracy of the four established prognostic models. **(H)** Comparison of the C-indices for the DEFRlncRNAs prognostic model with those of the other four prognostic models. **(I-J)** Univariate and multivariate Cox regression analyses examining the relationship between risk scores and clinical characteristics of CRC patients. **(K)** Performance of the prediction model across early (I-II) and late (III-IV) clinical stages.

Using the 44 DEFRlncRNAs identified from univariate Cox regression analysis, we further applied multivariate Cox regression to pinpoint 15 DEFRlncRNAs significantly associated with the prognosis of CRC patients (**Figure 2B**). Based on the median risk score, patients were classified into low-risk and high-risk groups (see **Supplementary Table S1** for clinicopathological information). A scatter plot illustrated the distribution of risk scores (**Figure 2C**) and the corresponding survival status, showing that most deaths occurred in the high-risk group (**Figure 2D**). Kaplan-Meier (KM) survival curves demonstrated that the overall survival (OS) of the high-risk group was significantly lower than that of the low-risk group (**Figure 2A**). The analysis of the area under the curve (AUC) for the risk model indicated that the AUCs for 1-, 3-, and 5-year survival were 0.840, 0.858, and 0.865, respectively (**Figure 2E**). Compared to age (AUC=0.598), gender (AUC=0.440), and clinical stage (AUC=0.707) in CRC patients, our risk model (AUC=0.840) demonstrated superior predictive accuracy for OS (**Figure 2F**). In addition, we compared the predictive ability of the DEFRlncRNAs model described in our study with that of four previously established prognostic models (8, 16–18). To ensure consistency and comparability, we applied the same method for calculating and converting the risk scores across the cohorts. The results indicated that the AUCs of our model were superior to those of the four previously published models (**Figure 2G**). Furthermore, the C-index for our model was the highest at 0.815, while the C-indices for the four other models were 0.653 (18), 0.575 (8), 0.654 (17), and 0.625 (16), respectively. These findings demonstrate that the prognostic performance of the DEFRlncRNAs signature consistently outperforms the other evaluated signatures (**Figure 2H**). Moreover, both univariate (**Figure 2I**) and multivariate Cox regression analyses (**Figure 2J**) confirmed that the risk score was an independent prognostic factor for poor outcomes in CRC patients (hazard ratio >1, p<0.05). To assess the model’s predictive performance across clinical stages, we evaluated its effectiveness in both early and late-stage patients. The results indicated that our model performs consistently well across all stages of CRC (**Figure 2K**). These findings suggest that our ferroptosis-related lncRNA risk model is a reliable prognostic tool for CRC patients.

### Characteristics of the DEFRlncRNAs prognostic model

**Figure 3:**
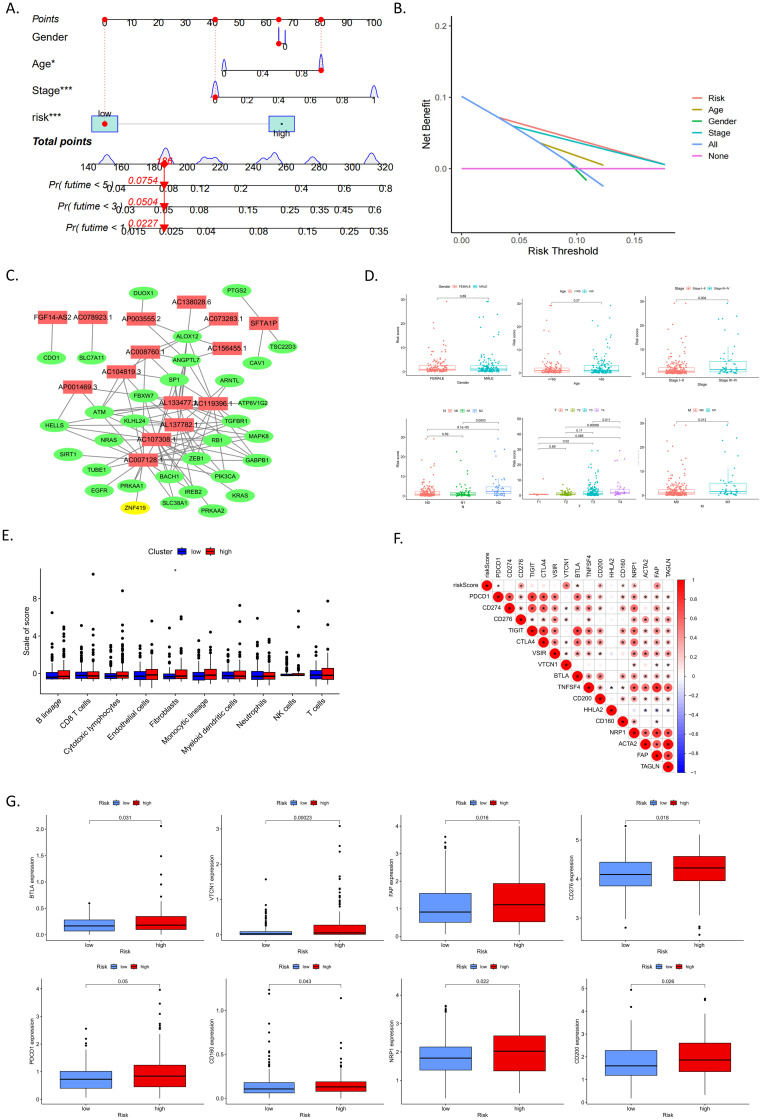
**(A)** Nomogram assigning points to each variable, allowing the calculation of a total score for each patient. The bottom scale predicts the probability of OS at specified time points based on this score. **(B)** Decision curve analysis (DCA) illustrating the clinical value of the nomogram model. **(C)** Interaction network of 15 DEFRlncRNAs (shown in red) and 32 interacting proteins. **(D)** Associations between risk scores and clinicopathological features, including age, gender, clinical stage, and TNM classification. **(E)** Correlations between risk score and the tumor microenvironment (TME). **(F)** Correlations between risk score and immune checkpoints as well as tumor-associated fibroblast markers. **(G)** Differential expression of immune checkpoints and tumor-associated fibroblast markers between high- and low-risk groups.

A reliable nomogram for predicting individual survival risk was constructed based on multiple regression analysis, identifying three variables—age, stage, and risk scores—with significant P values <0.05 (**Figure 3A**). Decision curve analysis (DCA) demonstrated that the DEFRlncRNAs prognostic model provided better predictive accuracy than other predictors (**Figure 3B**). We visualized an interaction network comprising 15 DEFRlncRNAs and 32 interacting proteins to elucidate their associations (**Figure 3C**). The relationship between the prognostic signature and clinicopathological features was examined by comparing risk scores across groups categorized by age, gender, clinical stage, and TNM classification. The analysis revealed that risk scores were unaffected by age or gender but significantly increased with advancing tumor stage and TNM classification (**Figure 3D**).

To understand the impact of our risk model on the tumor microenvironment (TME), we estimated the abundance of various immune and stromal cell populations in CRC tissue. Results showed that the high-risk group in our model had a higher abundance of fibroblasts (**Figure 3E**). Although there were no differences in the abundance of tumor-infiltrating immune cells between high and low-risk groups, we analyzed the correlation between risk scores and tumor-associated fibroblast markers as well as immune checkpoints (**Figure 3F**). The expression levels of immune checkpoints, including BTLA, VTCN1, CD276, PDCD1, CD160, NRP2, and CD200, as well as the tumor-associated fibroblast marker FAP, were found to be higher in the high-risk group (**Figure 3G**). These results suggest that the prognostic signature based on DEFRlncRNAs may have potential in indicating immune status and predicting patient response to immunotherapy.

### Functions of prognosis-related DEFRlncRNAs in colorectal cancer

**Figure 4:**
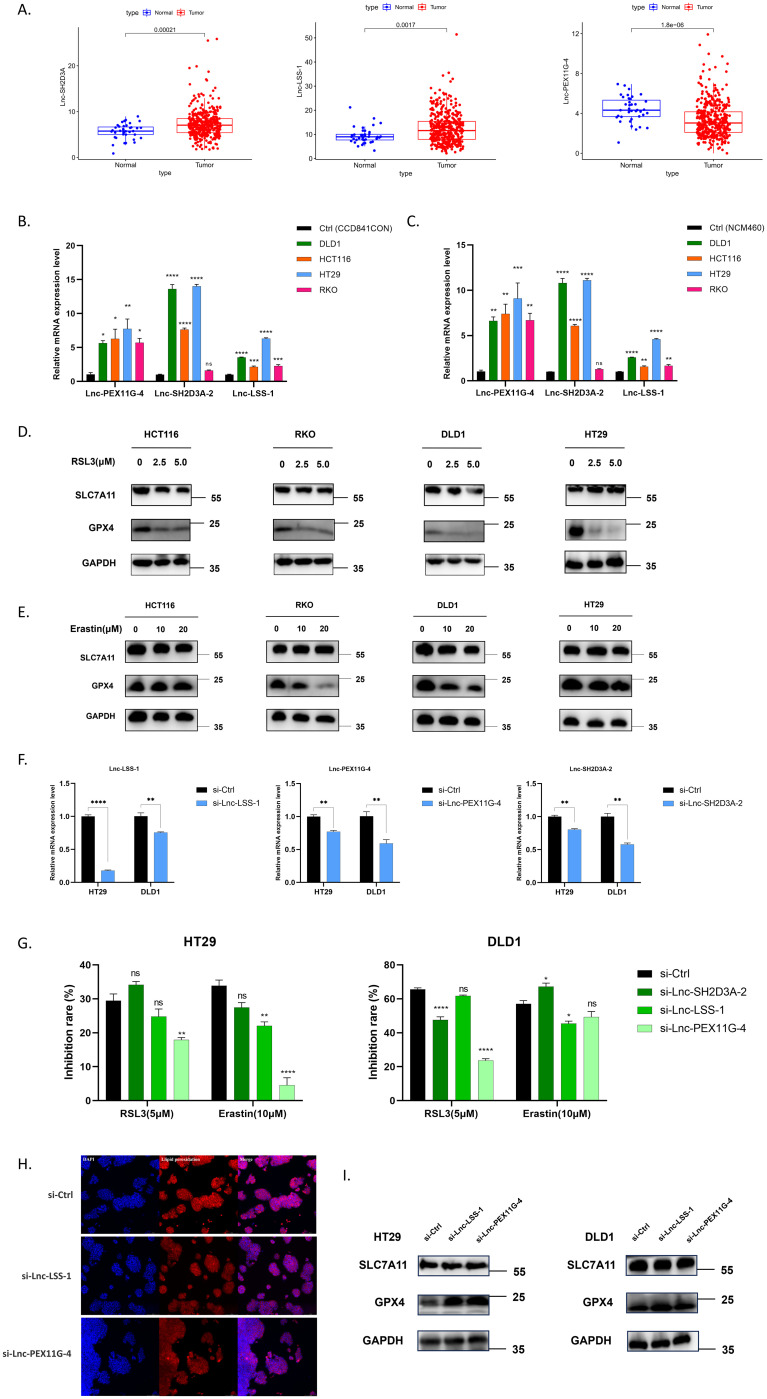
**(A)** Expression levels of Lnc-SH2D3A-2, Lnc-LSS-1, and Lnc-PEX11G-4 in the TCGA CRC database. **(B, C)** qPCR validation of the relative expression of Lnc-SH2D3A-2, Lnc-LSS-1, and Lnc-PEX11G-4 in various human CRC cell lines compared to normal colonic epithelial cell lines CCD841CON and NCM460. **(D, E)** Changes in protein levels of key ferroptosis markers GPX4 and SLC7A11 in CRC cell lines treated with varying concentrations of the ferroptosis agonists RSL3 and Erastin for 48 hours. **(F)** qPCR validation of siRNA knockdown efficiency for Lnc-LSS-1, Lnc-PEX11G-4, and Lnc-SH2D3A-2 in HT29 and DLD1 cell lines. **(G)** CCK-8 assay results showing the inhibition of ferroptosis in CRC cell lines upon knockdown of Lnc-LSS-1 and Lnc-PEX11G-4, when induced by ferroptosis agonists. **(H)** Immunofluorescence detection of lipid peroxidation levels, indicated by fluorescence intensity, in CRC cell lines after knockdown of Lnc-LSS-1 and Lnc-PEX11G-4. **(I)** Western blot analysis showing that knockdown of Lnc-LSS-1 and Lnc-PEX11G-4 reduces ferroptosis in CRC cells primarily through the regulation of GPX4 expression, rather than SLC7A11.

To explore the biological functions of 15 prognosis-related DEFRlncRNAs in CRC, we used the TCGA database and select 2 lncRNAs (AC008760.1 and AP001469.3) with high basal expression levels and 1 lncRNA (AC104819.3) with low basal expression level (**Figure 4A**). These correspond to Lnc-SH2D3A-2, Lnc-LSS-1, and Lnc-PEX11G-4, respectively (see **Supplementary Table S2** for all 15 DEFRlncRNAs’ LNCipedia gene IDs). We first assessed the expression levels of these lncRNAs in normal colonic epithelial cell lines (CCD841CON [**Figure 4B**] and NCM460 [**Figure 4C**]) compared to four CRC cell lines (RKO, HCT116, HT29, DLD1). qPCR results showed that Lnc-LSS-1, Lnc-PEX11G-4, and Lnc-SH2D3A-2 were significantly upregulated in HCT116, HT29, and DLD1 cells compared to normal colonic epithelial cells (primer sequences in **Supplementary Table S3**). To investigate the involvement of these DEFRlncRNAs in CRC ferroptosis, we treated the four CRC cell lines with ferroptosis agonists RSL3 (**Figure 4D**) and Erastin (**Figure 4E**). Our results indicated that RKO, HT29, and DLD1 cells were sensitive to these agonists, while HCT116 cells were relatively resistant. Based on the basal expression levels of the three DEFRlncRNAs and cell line sensitivity to ferroptosis agonists, we selected HT29 and DLD1 for further studies.

We generated HT29 and DLD1 cell lines with knockdown of Lnc-LSS-1, Lnc-PEX11G-4, and Lnc-SH2D3A-2 using specific siRNAs, confirming the knockdown efficiency by qPCR (**Figure 4F**). To evaluate the impact of DEFRlncRNAs knockdown on CRC, we evaluated cell viability after treating these cells with ferroptosis agonists. Knockdown of Lnc-PEX11G-4 significantly inhibited ferroptosis induced by RSL3 and Erastin in HT29 cells, whereas knockdown of Lnc-LSS-1 exhibited a trend of inhibiting ferroptosis in both DLD1 and HT29 cells (**Figure 4G**). However, when Lnc-SH2D3A-2 was knocked down, the ferroptosis induction followed a different trend. This could be attributed to Lnc-SH2D3A-2’s relatively minor role in regulating ferroptosis, or potentially to compensation by other redundant mechanisms. As a result, we decided to focus on the knockdown experiments of Lnc-LSS-1 and Lnc-PEX11G-4 in our subsequent research. Immunofluorescence staining was performed to further investigate the effect of DEFRlncRNAs knockdown on lipid peroxidation, a key marker of ferroptosis. As illustrated in **Figure 4H**, knockdown of Lnc-LSS-1 and Lnc-PEX11G-4 effectively reduced lipid peroxidation levels in CRC, as indicated by decreased fluorescence intensity of the lipid peroxidation marker. To elucidate the molecular mechanisms underlying the inhibition of ferroptosis, we conducted Western blot analysis for key ferroptosis-related proteins. The results, presented in **Figure 4I**, suggest that knockdown of Lnc-LSS-1 and Lnc-PEX11G-4 reduces ferroptosis in CRC cells primarily through the regulation of GPX4 expression, rather than SLC7A11.

## Discussion and conclusions

Ferroptosis, a novel form of cell death, has garnered considerable attention in CRC research in recent years. Characterized by the accumulation of iron ions and ROS, leading to lipid peroxidation and subsequent damage to the cell membrane (19). The induction of ferroptosis has been demonstrated to infect tumor cell growth and survival, offering a promising new strategy for CRC treatment (20, 21). For instance, research by Sui et al. showed that RSL3 induces ferroptosis in CRC through GPX4 inactivation and ROS generation (22). Additionally, Xu et al. found that targeting SLC7A11 impairs the stemness of CRC cells, thereby inducing ferroptosis (23). The exploration of genes and their regulatory mechanisms involved in ferroptosis in CRC, along with the construction of prognostic models, is expected to have significant clinical implications for patient treatment.

Long non-coding RNAs (lncRNAs), as key regulators of gene expression, play a crucial role in the ferroptosis process in CRC. By modulating genes related to iron metabolism and oxidative stress, lncRNAs influence the ferroptosis pathway. For example, Qu et al. highlighted the role of lncRNAs in iron metabolism, ferroptosis, and cancer (11). Moreover, Yang et al. discovered that lncRNA-CBSLR affects CRC cell proliferation and apoptosis by regulating CBSLR/YTHDF2/CBS signaling axis (24). Similarly, research by Zhang et al. demonstrated that lncRNA-PMAN promotes the cytoplasmic translocation of ELAVL1 in peritoneal dissemination to inhibit ferroptosis (25). These findings suggest that lncRNAs may have a significant and potentially pivotal role in regulating ferroptosis within CRC.

In this study, we performed a comprehensive analysis to clarify the prognostic significance and biological roles of ferroptosis-related lncRNAs (DEFRlncRNAs) in CRC. Utilizing data from the TCGA database, we identified 15 DEFRlncRNAs significantly associated with CRC prognosis. Our prognostic model, based on the expression profiles of these lncRNAs, demonstrated superior predictive accuracy for OS compared to traditional clinical factors such as age, gender, and clinical stage. Among the 15 identified DEFRlncRNAs, 3 lncRNAs that could not be mapped to LNCipedia gene IDs (AL133477.1, AC119396.1, AL137782.1) and 7 lncRNAs (lnc-MLXIP-12, lnc-RNF166-1, lnc-KRR1-4, lnc-MSRB3-7, lnc-EPCAM-6, lnc-FGF3-1, lnc-NXPH1-2) were excluded due to their extremely low baseline expression levels, leaving us with 5 DEFRlncRNAs for further analysis. Among these, previous studies have shown that lnc-USP6NL-7 is regulated by the YAP/TAZ/TEAD signaling pathway in non-small cell lung cancer (NSCLC), where it enhances this pathway’s activity through a positive feedback mechanism, thereby promoting tumor cell proliferation and survival (26). Moreover, the downregulation of lnc-USP6NL-7 has also been shown to inhibit tumor cell proliferation and induce cell cycle arrest at the S phase, potentially through the PI3K-AKT signaling pathway (27). Additionally, lnc-USP6NL-7 has been reported to be highly expressed in hepatocellular carcinoma, where it promotes tumor growth by activating the PI3K/AKT/mTOR pathway via the suppression of miR-4766-5p expression (28). Furthermore, FGF14-AS2 has been implicated in glioma tumorigenesis by forming a feedback loop with the miR-320a/E2F1 axis (29). In breast cancer, FGF14-AS2 suppresses metastasis through the regulation of the miR-370-3p/FGF14 axis (30), and in CRC, it is believed to influence tumor progression via the PI3K/AKT/mTOR signaling pathway (31). However, the functions of Lnc-SH2D3A-2, Lnc-LSS-1, and Lnc-PEX11G-4 remain uncharacterized in the current literature.

We further identified three DEFRlncRNAs (Lnc-SH2D3A-2, Lnc-LSS-1, and Lnc-PEX11G-4), that were significantly upregulated in CRC cell lines compared to normal colonic epithelial cells. Notably, while public database data indicated lower RNA levels of Lnc-PEX11G-4 in CRC tissues compared to normal tissues, our results showed significantly elevated Lnc-PEX11G-4 expression in CRC cell lines relative to normal colonic epithelial cells. This discrepancy may stem from several factors (32). Public database data often include diverse patient samples with various cell types, whereas our experiments focused specifically on selected cell lines, providing a more precise reflection of gene expression in cancer cells. Additionally, data from public databases might be influenced by varying experimental conditions and data processing methods, while qPCR offers higher sensitivity and specificity, accurately quantifying gene expression in specific cells. Moreover, the growth conditions of cell lines *in vitro* differ from the *in vivo* tumor microenvironment, potentially contributing to expression differences (33). Therefore, although public databases offer valuable insights into gene expression patterns, our qPCR results provide more direct and specific evidence that Lnc-PEX11G-4 is markedly upregulated in CRC cells compared to normal colonic epithelial cells.

Functional assays revealed that knockdown of Lnc-LSS-1 and Lnc-PEX11G-4 inhibited ferroptosis induced by ferroptosis agonists RSL3 and Erastin in HT29 and DLD1 cells, suggesting these lncRNAs play a critical role in regulating ferroptosis in CRC. Immunofluorescence staining and Western blot analysis provided further insights into the molecular mechanisms underlying this inhibition. Knockdown of Lnc-LSS-1 and Lnc-PEX11G-4 led to a reduction in lipid peroxidation levels and primarily regulated GPX4 expression, a key enzyme in the ferroptosis pathway (34, 35), without significantly affecting SLC7A11 (36). This highlights the specific regulatory pathways through which these lncRNAs influence ferroptosis in CRC cells.

The implications of our findings are significant for CRC research and treatment. The ability of DEFRlncRNAs to predict patient prognosis and influence ferroptosis suggests they could serve as valuable biomarkers and therapeutic targets. Incorporating the expression profiles of DEFRlncRNAs into clinical decision-making could enhance the accuracy of CRC prognosis and enable more personalized treatment strategies, especially given the limitations of the current TNM staging system. Moreover, targeting ferroptosis presents a promising therapeutic strategy for CRC. Our study demonstrates that modulating the expression of specific lncRNAs can influence ferroptosis and potentially suppress tumor growth. This approach could be combined with existing therapies to enhance their efficacy and overcome resistance mechanisms. Future research should focus on validating these findings in larger, independent cohorts and exploring the therapeutic potential of DEFRlncRNAs in clinical settings.

In summary, our study provides compelling evidence for the prognostic and functional significance of DEFRlncRNAs in CRC. By identifying and characterizing these lncRNAs, we offer new insights into the molecular mechanisms underlying CRC progression and ferroptosis. These findings pave the way for the development of novel prognostic models and therapeutic strategies, ultimately improving clinical outcomes for CRC patients.

## Data Availability

The original contributions presented in the study are included in the article/**Supplementary Material**. Further inquiries can be directed to the corresponding authors.

## References

[1] Siegel RL Miller KD Wagle NS Jemal A. Cancer statistics, 2023. CA Cancer J Clin. (2023) 73:17–48. doi: 10.3322/caac.21820 36633525

[2] Ding X Huang H Fang Z Jiang J. From subtypes to solutions: integrating CMS classification with precision therapeutics in colorectal cancer. Curr Treat Opt Oncol. (2024) 25:1580–93. doi: 10.1007/s11864-024-01282-5 39589648

[3] Jiang X Stockwell BR Conrad M. Ferroptosis: mechanisms, biology and role in disease. Nat Rev Mol Cell Biol. (2021) 22:266–82. doi: 10.1038/s41580-020-00324-8 PMC8142022 33495651

[4] Yang J Mo J Dai J Ye C Cen W Zheng X Jiang L. Cetuximab promotes RSL3-induced ferroptosis by suppressing the Nrf2/HO-1 signalling pathway in KRAS mutant colorectal cancer. Cell Death Dis. (2021) 12:1079. doi: 10.1038/s41419-021-04367-3 34775496 PMC8590697

[5] Adamiec-Organisciok M Wegrzyn M Cienciala L Sojka D Nackiewicz J Skonieczna M. Compensative resistance to erastin-induced ferroptosis in GPX4 knock-out mutants in HCT116 cell lines. Pharmaceut (Basel). (2023) 16. doi: 10.3390/ph16121710 PMC10747702 38139836

[6] Winkler L Dimitrova N. A mechanistic view of long noncoding RNAs in cancer. Wiley Interdiscip Rev RNA. (2022) 13:e1699. doi: 10.1002/wrna.1699 34668345 PMC9016092

[7] Chen LJ Chen X Niu XH Peng XF. LncRNAs in colorectal cancer: Biomarkers to therapeutic targets. Clin Chim Acta. (2023) 543:117305. doi: 10.1016/j.cca.2023.117305 36966964

[8] Pang L Wang Q Wang L Hu Z Yang C Li Y. Development and validation of cuproptosis-related lncRNA signatures for prognosis prediction in colorectal cancer. BMC Med Genomics. (2023) 16:58. doi: 10.1186/s12920-023-01487-x 36949429 PMC10031908

[9] Islam MS Gopalan V Lam AK Shiddiky MJA. Current advances in detecting genetic and epigenetic biomarkers of colorectal cancer. Biosens Bioelectr. (2023) 239:115611. doi: 10.1016/j.bios.2023.115611 37619478

[10] Andrabi MQ Kesavan Y Ramalingam S. Non-coding RNAs as biomarkers for survival in colorectal cancer patients. Curr Aging Sci. (2024) 17:5–15. doi: 10.2174/1874609816666230202101054 36733201

[11] Qu L He X Tang Q Fan X Liu J Lin A. Iron metabolism, ferroptosis, and lncRNA in cancer: knowns and unknowns. J Zhejiang Univ Sci B. (2022) 23:844–62. doi: 10.1631/jzus.B2200194 PMC9561407 36226538

[12] Wu HH Zhang W Chang J Wu J Zhang X Jia F. Comprehensive analysis of mitochondrial-related gene signature for prognosis, tumor immune microenvironment evaluation, and candidate drug development in colon cancer. Sci Rep. (2025) 15:6173. doi: 10.1038/s41598-024-85035-2 39979377 PMC11842742

[13] Hu H Liu M Zeng Z. Penalized Lq-likelihood estimator and its influence function in generalized linear models. Metrika. (2025) 88:1–18. doi: 10.1007/s00184-023-00943-z

[14] Liao RG Wang JH Zhang F Fang YT Zhou L Zhang YQ. A novel mitochondrial-related risk model for predicting prognosis and immune checkpoint blockade therapy response in uterine corpus endometrial carcinoma. Sci Rep. (2025) 15:1404. doi: 10.1038/s41598-025-85537-7 39789247 PMC11717914

[15] Zhu D Zhang X Fang Y Xu Z Yu Y Zhang L. Identification of a lactylation-related gene signature as the novel biomarkers for early diagnosis of acute myocardial infarction. Int J Biol Macromol. (2024) 282:137431. doi: 10.1016/j.ijbiomac.2024.137431 39521235

[16] Xiao L Yin W Chen X Zhang X Zhang C Yu Z. A disulfidptosis-related lncRNA index predicting prognosis and the tumor microenvironment in colorectal cancer. Sci Rep. (2023) 13:20135. doi: 10.1038/s41598-023-47472-3 37978247 PMC10656577

[17] Thandar M Zhu Y Zhang X Chen Z Zhao Y Huang S. Construction and validation of stemness-related lncRNA pair signature for predicting prognosis in colorectal cancer. J Cancer Res Clin Oncol. (2023) 149:11815–28. doi: 10.1007/s00432-023-05047-9 PMC11798197 37410143

[18] Dong C Guo Y Wang P Yin S Ge X. Comprehensive analysis of disulfidptosis-related lncRNA features for prognosis and immune landscape prediction in colorectal cancer. Front Oncol. (2023) 13:1287808. doi: 10.3389/fonc.2023.1287808 38213838 PMC10783935

[19] Yang WS Stockwell BR. Ferroptosis: death by lipid peroxidation. Trends Cell Biol. (2016) 26:165–76. doi: 10.1016/j.tcb.2015.10.014 PMC4764384 26653790

[20] Chen X Kang R Kroemer G Tang D. Broadening horizons: the role of ferroptosis in cancer. Nat Rev Clin Oncol. (2021) 18:280–96. doi: 10.1038/s41571-020-00462-0 33514910

[21] Singhal R Mitta SR Das NK Kerk SA Sajjakulnukit P Solanki S. HIF-2alpha activation potentiates oxidative cell death in colorectal cancers by increasing cellular iron. J Clin Invest. (2021) 131. doi: 10.1172/JCI143691 PMC8203462 33914705

[22] Sui X Zhang R Liu S Duan T Zhai L Zhang M. RSL3 drives ferroptosis through GPX4 inactivation and ROS production in colorectal cancer. Front Pharmacol. (2018) 9:1371. doi: 10.3389/fphar.2018.01371 30524291 PMC6262051

[23] Xu X Zhang X Wei C Zheng D Lu X Yang Y. Targeting SLC7A11 sp*ecifically suppresses the progression of colorectal cancer stem cells via inducing ferroptosis* . Eur J Pharm Sci. (2020) 152:105450. doi: 10.1016/j.ejps.2020.105450 32621966

[24] Yang H Hu Y Weng M Liu X Wan P Hu Y. Hypoxia inducible lncRNA-CBSLR modulates ferroptosis through m6A-YTHDF2-dependent modulation of CBS in gastric cancer. J Adv Res. (2022) 37:91–106. doi: 10.1016/j.jare.2021.10.001 35499052 PMC9039740

[25] Lin Z Song J Gao Y Huang S Dou R Zhong P. Hypoxia-induced HIF-1alpha/lncRNA-PMAN inhibits ferroptosis by promoting the cytoplasmic translocation of ELAVL1 in peritoneal dissemination from gastric cancer. Redox Biol. (2022) 52:102312. doi: 10.1016/j.redox.2022.102312 35447413 PMC9043498

[26] Zhu B Finch-Edmondson M Leong KW Zhang X V M Lin QXX. LncRNA SFTA1P mediates positive feedback regulation of the Hippo-YAP/TAZ signaling pathway in non-small cell lung cancer. Cell Death Discov. (2021) 7:369. doi: 10.1038/s41420-021-00761-0 34845189 PMC8630011

[27] Du D Shen X Zhang Y Yin L Pu Y Liang G. Expression of long non-coding RNA SFTA1P and its function in non-small cell lung cancer. Pathol Res Pract. (2020) 216:153049. doi: 10.1016/j.prp.2020.153049 32825934

[28] Huang G Yang Y Lv M Huang T Zhan X Kang W. Novel lncRNA SFTA1P Promotes Tumor Growth by Down-Regulating miR-4766-5p via PI3K/AKT/mTOR Signaling Pathway in Hepatocellular Carcinoma. Onco Targets Ther. (2020) 13:9759–70. doi: 10.2147/OTT.S248660 PMC7533222 33061455

[29] Zhang P Gu X Zhang N Liu L Dong X Li H. FGF14-AS2 accelerates tumorigenesis in glioma by forming a feedback loop with miR-320a/E2F1 axis. J Cancer. (2021) 12:6429–38. doi: 10.7150/jca.62120 PMC8489148 34659533

[30] Jin Y Zhang M Duan R Yang J Yang Y Wang J. Long noncoding RNA FGF14-AS2 inhibits breast cancer metastasis by regulating the miR-370-3p/FGF14 axis. Cell Death Discov. (2020) 6:103. doi: 10.1038/s41420-020-00334-7 33083023 PMC7548970

[31] Su T Huang L Zhang N Peng S Li X Wei G. FGF14 functions as a tumor suppressor through inhibiting PI3K/AKT/mTOR pathway in colorectal cancer. J Cancer. (2020) 11:819–25. doi: 10.7150/jca.36316 PMC6959027 31949485

[32] Pinkney HR Ross CR Hodgson TO Pattison ST Diermeier SD. Discovery of prognostic lncRNAs in colorectal cancer using sp*atial transcriptomics* . NPJ Precis Oncol. (2024) 8:230. doi: 10.1038/s41698-024-00728-1 39390212 PMC11467462

[33] Suliman M Saleh RO Chandra M Rasool KH Jabir M Jawad SF. Macrophage-derived lncRNAs in cancer: regulators of tumor progression and therapeutic targets. Med Oncol. (2025) 42:91. doi: 10.1007/s12032-025-02643-2 40048034

[34] Zhang W Liu Y Liao Y Zhu C Zou Z. GPX4, ferroptosis, and diseases. BioMed Pharmacother. (2024) 174:116512. doi: 10.1016/j.biopha.2024.116512 38574617

[35] Liu Y Wan Y Jiang Y Zhang L Cheng W. GPX4: The hub of lipid oxidation, ferroptosis, disease and treatment. Biochim Biophys Acta Rev Cancer. (2023) 1878:188890. doi: 10.1016/j.bbcan.2023.188890 37001616

[36] Chen X Li J Kang R Klionsky DJ Tang D. Ferroptosis: machinery and regulation. Autophagy. (2021) 17:2054–81. doi: 10.1080/15548627.2020.1810918 PMC8496712 32804006

