# Supplementary material for: Identification and functional characterization of prognosis-related ferroptosis-associated lncRNAs in colorectal cancer
Source: Front Immunol. 2025 Apr 29;16:1561210. doi: 10.3389/fimmu.2025.1561210 (PMC12069887; doi:10.3389/fimmu.2025.1561210)
Supplement: Supplementary file 4 [file Table4.docx]

Figure1 raw data：https://www.jianguoyun.com/p/DVw47TEQzrCZDRiWmPQFIAA (访问密码 : ctgu08)

Figure2 raw data：https://www.jianguoyun.com/p/DQT0C0oQzrCZDRidmPQFIAA (访问密码 : ax90tm)

Figure3 raw data：https://www.jianguoyun.com/p/DQ7-4w4QzrCZDRihmPQFIAA (访问密码 : 76zlgj)

Figure4 raw data：https://www.jianguoyun.com/p/DQLBY_cQzrCZDRijmPQFIAA (访问密码 : yg154h)
